# Perceptions of the local beekeepers on the diversity and flowering phenology of the melliferous flora in the community of Xmabén, Hopelchén, Campeche, Mexico

**DOI:** 10.1186/s13002-019-0296-1

**Published:** 2019-03-08

**Authors:** Milca E. Coh-Martínez, William Cetzal-Ix, Jesús F. Martínez-Puc, Saikat Kumar Basu, Eliana Noguera-Savelli, Manuel J. Cuevas

**Affiliations:** 1 Tecnológico Nacional de México, Instituto Tecnológico de Chiná, Calle 11 entre 22 y 28 Colonia Centro Chiná, 24050 Campeche, Mexico; 2 Department of Biological Sciences, University of Lethbridge, Lethbridge, AB T1K 3M4 Canada; 3 Catedrática CONACYT, Colegio de Postgraduados Campus Campeche, Carretera Haltunchén-Edzná Km. 17.5, Sichochac, Champotón, Campeche, Mexico

**Keywords:** Beekeeping cycle, Indigenous knowledge, Melliferous flora, Native plants, Yucatan peninsula

## Abstract

**Background:**

The knowledge of native melliferous flora (MF) may contribute to identify the diversity of species available for beekeeping activities during the dry and rainy seasons of the year in the Yucatan Peninsula (YP) region. The acute shortage of food resources considerably reduce local honey production and needs to be addressed appropriately. The objective of this study has been identifying the local MF, their nectar and pollen contribution, their flowering patterns, and the criteria of the vegetation to be established adjacent to local apiaries for stable production of quality honey. The study also investigates how this approach helps to complete the annual flowering cycle required to maintain the honeybee colonies, thereby preventing swarm escapes during periods of acute food stress in the community of Xmabén, Hopelchén, Campeche, Mexico.

**Methodology:**

We conducted a comprehensive survey based on interviews with 40 local beekeepers and a review of herbarium specimens (CICY) of the database of the global information network on the native MF biodiversity with high apiculture potential, the contribution of nectar and pollen they provide, and their flowering patterns. Furthermore, we documented interviews with the same beekeepers on the necessary conditions for establishing the ideal components of vegetation in areas adjacent to apiaries for high-quality honey production in the Xmabén community of Hopelchén, Campeche, Mexico.

**Results:**

We have identified 56 native MF species with apiculture potential, that need to be planted around the apiaries for assisting honeybees in successfully running the beekeeping production cycle. Hence, the MF diversity of Xmabén community constitutes a valuable resource for successful beekeeping in the region and adjoining localities. We found that 22.5% of local beekeepers are dedicated exclusively to apilcilture, while 77.5% practice it as a secondary activity due to better sources of income in agriculture (60%), masonry (10%), and livestock management (7.5%). The data generated can help in further expansion of the local apiaries, beekeeping business, and in building future opportunities for the local apiculture industry. Indigenous knowledge of the beekeepers was comprehensive and corroborated the technical information on MF collected from the herbarium, further emphasizing the value of indigenous knowledge on traditional beekeeping practices.

**Conclusion:**

From the perspective of human ecology, our study reveals the need of collecting, analyzing, and interpreting indigenous knowledge to facilitate traditional beekeeping practices of the region without using expensive, modern technology to solve ecosystem-based problems through long-term, sustainable, traditional, and environment friendly approaches.

## Background

In Mexico, beekeeping is one of the main economic activities in the agricultural sector, with an average honey production of 57,000 tons per year, ranking sixth in exports worldwide [1]. The country is divided into five distinct apicultural regions [2]. The Yucatan Peninsula (YP) is the most important honey-producing region since it is home to 30–35% of the local honeybee (*Apis melifera* L.) colonies, and exports 80–95% of the total Mexican honey produced to different international markets [3, 4]. The YP is constituted by the states of Campeche, Yucatan, and Quintana Roo; the Campeche state occupied the first place in national honey production, with its main producing municipalities being Champotón, Campeche, and Calkiní, followed by Hopelchén, Hecelchakán, and Tenabo [5].

The YP honey is appreciated in the international markets for its organoleptic characteristics (color, aroma, and taste) that depend upon the specific biotic and abiotic conditions of the region [6]. However, the production of honey based on the availability of nectar and pollen resources vary depending on the local vegetation type throughout the year [7]. According to Velázquez-Rentería [6], a total of 900 species of flowering plants provide nectar and/or pollen to the honeybees in the YP. This diversity of melliferous plants represents 38% of the flora of YP, considering that there are about 2329 taxa in 956 genera and 161 families of native or naturalized plants [8]. Although there is a great diversity of melliferous flora (MF), only a select group of common honey species are known and used in the YP [9]. Nearly 90% of the annual production of honey comes from the nectar flow of *Viguiera dentata* (Cav.) Spreng (42%, flowering between December and February) and *Gymnopodium floribundum* Rolfe (48%, flowering between March and May); the remaining 10% comes from the nectar flow of legumes (Fabaceae) and climber species of Sapindaceae, Convolvulaceae plant families [7].

The information of MF with common species useful for beekeeping is available in previously cited regional studies [6–9]; however, there is a shortage of research on MF at the level of municipalities, communities, or localities related with information of annual seasons or the beekeeping cycle in the PY. For example, the YP beekeeping cycle has unique relationships with the dry and rainy season (amount of precipitation), that in turn influence the flowering periods of the species that provide nectar and/or pollen for feeding the bees and facilitate the sustenance of their colonies [10, 11]. The beekeeping cycle is divided into three stages: harvest (January to May), post-harvest (June to September), and pre-harvest (October to December) [12]. The harvest stage occurs in the driest period of the year, when the greatest diversity of honey plants (melliferous) bloom and the highest honey production is reported [7, 12]. The post-harvest stage represents the rainy season, and the honey produced during this season has a high degree of humidity that affects the quality and its corresponding price [10, 11]. Furthermore, the rainy season marks the acute shortage of foraging flowering plants for the honeybees due to the reduced availability of flowers of melliferous species, thereby impacting honey production in the region [13].

The experiences, challenges, problems, and practices as depicted by the local beekeepers of a specific area about of MF; when they are validated with interviews or painstaking field study, this indigenous knowledge can represent a useful tool for the beekeeping of a region, modern bee researchers, and melliferous flora investigators. This case study presumes that knowledge on the native MF may contribute to identify the diversity of species available for the beekeeping activities during the dry and rainy seasons of the year in the YP region. The acute shortage of food resources reduce local honey production considerably and needs to be addressed appropriately. It is therefore important to establish relevant, comprehensive strategies for targeted enrichment and assemblages of MF in the surrounding areas of the local apiaries. Such an initiative can thereby help to complete the annual flowering cycle required to maintain the honeybee colonies, avoiding swarm escapes during periods of acute food stress. Such an approach has the potential to increase honey production in the local apiaries and improve the economic conditions of the families that are directly and indirectly dependent on the apiculture industry. This study aimed at identifying the local MF, the contribution of nectar and pollen they can provide, their flowering patterns, and the criteria of the vegetation to be established in and around local apiaries for stable production of quality honey, and successfully combating food shortage challenges for the local honeybees in the community of Xmabén, Hopelchén, Campeche, Mexico.

## Methods

### Study area

**Fig. 1:**
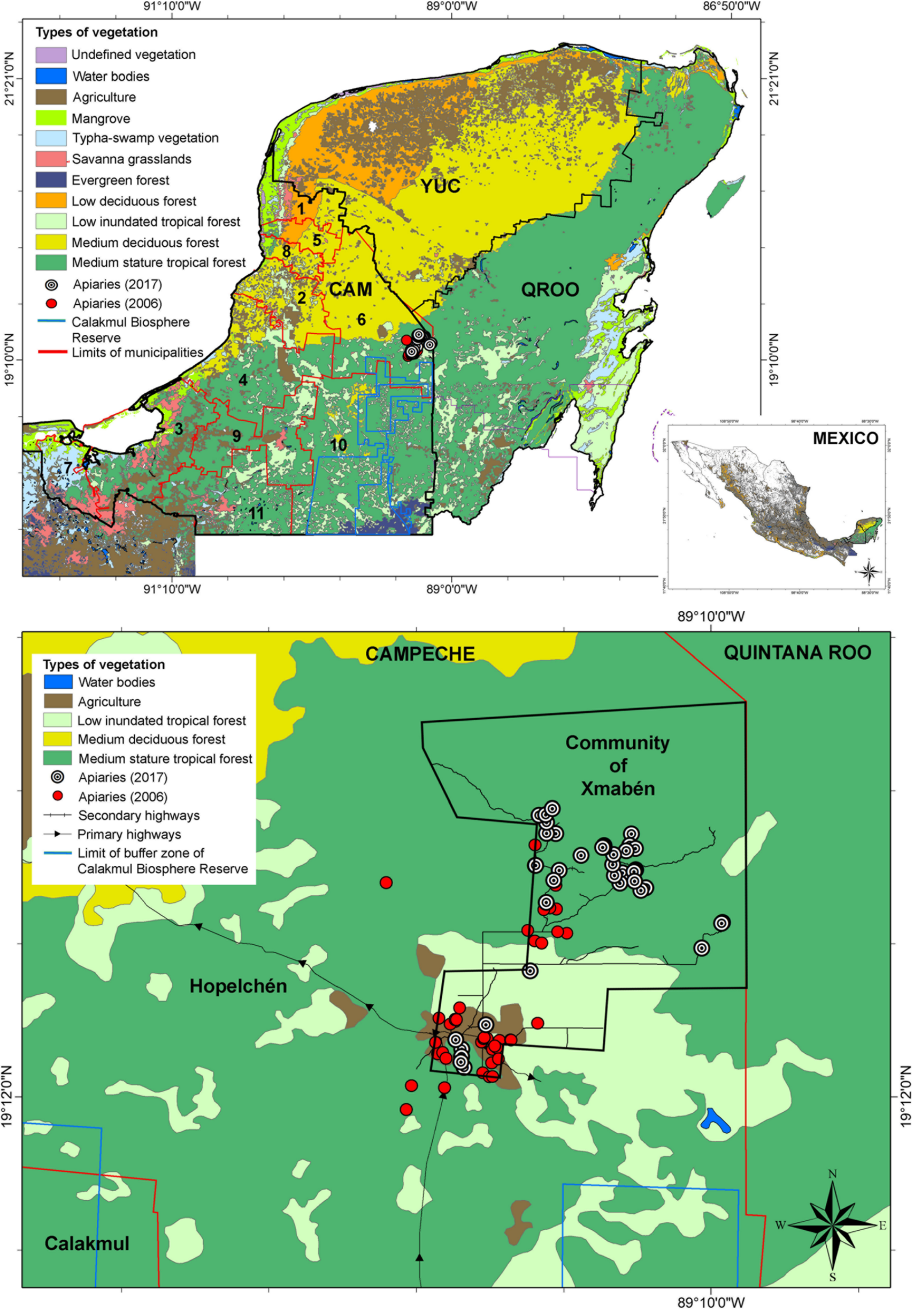
Study area, types of vegetation, and apiaries in the community of Xmabén, Hopelchén, Campeche, Mexico. Apiaries (red circles) and polygon layer of Xmabén based on Porter-Bolland and Ellis [16, 39], apiaries represented with black and white circles based on this study. States of Yucatan Peninsula (CAM = Campeche, QROO = Quintana Roo, YUC = Yucatán), Municipalities of Campeche (1 = Calkiní, 2 = Campeche, 3 = Carmen, 4 = Champotón, 5 = Hecelchakán, 6 = Hopelchén, 7 = Palizada, 8 = Tenabo, 9 = Escárcega, 10 = Calakmul, 11 = Candelaria)

The interviews of the beekeepers were conducted during 2017 in the community of Xmabén (89° 06′ and 90° 09′ W, 17° 48′ and 20° 11′ N), located in the municipality of Hopelchén, eastern Campeche, Mexico. Our study site is located in the central part of the YP at the convergence point of the states of Yucatán, Campeche, and Quintana Roo (Fig. 1). The community has an area of 49,680 ha, of which 5000 ha have been ceded to the Mennonites for 30 years, 2000 ha are used for agriculture, 2700 ha for livestock, 148 ha for urban areas, 25,800 ha for forestry, and 5060 ha for other uses [14]. The community has a population of 1300 inhabitants (only 216 are *ejidatarios* or those who own agrarian rights on their lands), and the main economic activities are subsistence agriculture, apiculture, and commercialization of chicle (*Manilkara zapota* (L.) P. Royen) [15]. Beekeeping is one of the most important economic activities due to the high demand for quality honey produced in the region and exported to various local, regional, and international markets [14].

### Beekeepers and apiaries

The interviews were conducted with 40 local beekeepers (100% of the people who perform this activity in the community), with open and closed questions. Initially, they were asked why they decided to dedicate themselves to this activity and their general data (sex, age, schooling, and main occupation). Subsequently, on technical aspects related to the number of apiaries, number of hives per apiary and government training for the establishment of apiaries. Additionally, the beekeepers were asked what are the criteria they use to establish their apiaries, whether they are permanent or static, private or ejido, the type of vegetation, and the time the apiary has been established. Furthermore, the location of individual apiary was recorded with global positioning system (GPS) and photographed with a Phantom Pro4 drone to identify vegetation types and access roads where the apiaries are established. We also used GPS data from community apiaries previously registered by Porter and Ellis [16]. Cartography was produced by plotting the localities of apiaries on an image of the vegetation types of CONABIO [17] using ArcView 3.2 [18].

### Melliferous flora

On the other hand, the interviews included open and closed questions about their familiarity and acquisition of indigenous knowledge on the local MF, the age at which they acquired this knowledge, the contribution of nectar and pollen the local MF provide, their flowering patterns, and the criteria to establish MF around the local apiaries for quality honey production during periods of acute food shortage for the local honeybees. On other hand, four permanent plots were established based on the proximity of the apiaries (circular of 1000 m^2^) in medium stature tropical forest (MSTF), low inundated tropical forest, and secondary vegetation or agriculture [17]. Monthly botanical collections and field observations were made in the surroundings of the community between February to December 2017, to identify the MF and corroborate the information provided by the local beekeepers. However, in this study, botanical collections were used only to corroborate the presence of species of MF; these specimens were deposited in the Universidad Autónoma de Campeche (UCAM) herbarium (Table 1). Additionally, several bibliographic sources were also consulted [8, 9, 12, 19] to confirm the specific uses of MF.

**Table 1:** List of melliferous flora in the community of Xmabén, Hopelchen, Campeche, Mexico

Family	Mayan name	Taxa	CN-N					Months											
			MECM	GH	N	P	N-P	J	F	M	A	M	J	J	A	S	O	N	D
Alismataceae	Kibix	*Sagittaria lancifolia* L. ssp. *media* (Micheli) Bogin	1	H			1										1	1	
Anacardiaceae	Boxchechen, Chechen negro	*Metopium brownei* (Jacq.) Urb. (*, +)	2	T			1	1	1	1			1	1					
Anacardiaceae	Joboo	*Spondias radlkoferi* Donn. Sm.	3	T			1			1		1	1						
Arecaceae	Bak allin	*Desmoncus orthacanthos* Mart.	4	L			1		1	1	1								
Asteraceae	Sak taj	*Koanophyllon albicaulis* (Sch. Bip. Ex Klatt) R. M. King & H. Rob.	5	S			1	1	1	1									1
Asteraceae	Tajonal	*Viguiera dentata* (Cav.) Spreng. var. *dentata* (*, +)	6	H			1	1	1										
Bignoniaceae	Jok ka	*Tabebuia rosea* (Bertol.) DC. (+)	7	T	1	1											1	1	
Bixaceae	Chu un	*Cochlospermum vitifolium* (Willd.) Spreng. (*, +)	8	S			1			1	1								
Boraginaceae	Bojon	*Cordia alliodora* (Ruiz & Pav.) Oken (*)	9	T			1	1	1	1	1								
Boraginaceae	Ciricote, kok che	*Cordia dodecandra* DC. (+)	10	T	1	1						1	1						
Burseraceae	Chakaj	*Bursera simaruba* (L.) Sarg. (*, +)	11	T	1	1		1	1	1	1								
Combretaceae	Puc tee	*Terminalia buceras* (L.) C. Wright	12	T			1	1	1	1	1							1	1
Convolvulaceae	Tsolen ak	*Jacquemontia oaxacana* (Meisn.) Hallier f.	13	C		1											1	1	1
Convolvulaceae	Tsots ak	*Jacquemontia pentantha* (Jacq.) G. Don (+)	14	C	1											1	1	1	
Convolvulaceae	Xtabentun	*Turbina corymbosa* (L.) Raf. (*, +)	15	C	1												1	1	1
Erythroxylaceae	Cascaron	*Erythroxylum confusum* Britton	16	T	1			1	1										
Euphorbiaceae	Xperes	*Croton arboreus* Millsp.	17	S			1	1	1	1	1	1							
Euphorbiaceae	Chul kej	*Croton niveus* Jacq.	18	S			1	1										1	1
Euphorbiaceae	Kok che	*Croton schiedeanus* Schltdl.	19	T	1							1	1						
Euphorbiaceae	Chechen blanco	*Sebastiania adenophora* Pax & K. Hoffm. (*)	20	T	1						1	1							
Fabaceae	Tsubin tul	*Acacia globulifera* Saff.	21	T	1						1	1							
Fabaceae	Kitin che	*Caesalpinia gaumeri* Greenm. (*, +)	22	T			1	1	1	1	1								
Fabaceae	Muk	*Dalbergia glabra* (Mill.) Standl. (+)	23	S			1										1	1	1
Fabaceae	Tsu tsul yuk	*Diphysa yucatanensis* Hanan-Alipi & M. Sousa	24	T	1				1	1	1								
Fabaceae	Tinto	*Haematoxylum campechianum* L. (+)	25	T			1	1	1										
Fabaceae	Vigas	*Lonchocarpus punctatus* Kunt (*, +)	26	T	1								1	1					
Fabaceae	Kan xuul	*Lonchocarpus xuul* Lundell (*, +)	27	T			1								1		1	1	
Fabaceae	Tsalan	*Lysiloma latisiliquum* (L.) Benth. (*, +)	28	T	1						1	1	1						
Fabaceae	Pica pica	*Mucuna pruriens* (L.) DC.	29	C		1									1	1	1	1	
Fabaceae	Jabin	*Piscidia piscipula* (L.) Sarg. (*, +)	30	T	1				1	1	1	1							
Fabaceae	Granadillo	*Platymiscium yucatanum* Standl. (+)	31	T	1			1	1	1	1								
Fabaceae	Kat sin	*Senegalia riparia* (Kunth) Britton & Rose	32	T		1											1	1	
Lamiaceae	Yax nik	*Vitex gaumeri* Greenm. (*, +)	33	T	1				1	1								1	1
Malvaceae	Puc(majagua)	*Hampea trilobata* Standl. (*, +)	34	T			1						1	1	1	1	1	1	
Malvaceae	Kaskat	*Luehea speciosa* Willd. (+)	35	T			1										1	1	1
Malvaceae	Malva	*Malvastrum coromandelianum* (L.) Garcke	36	H	1												1	1	
Myrtaceae	Guayavillo (Pichi che)	*Eugenia capuli* (Schlech. & Cham.) Hook & Arn. var. *capuli*	37	T	1							1	1						
Nyctaginaceae	Taj tsi	*Neea choriophylla* Standl.	38	T	1							1	1						
Nyctaginaceae	Beeb	*Pisonia aculeata* L.	39	L			1	1											
Picramniaceae	Chik che	*Picramnia antidesma* Sw.	40	S	1							1	1						
Poaceae	Tok suk	*Lasiacis grisebachii* (Nash) Hitchc. var. *grisebachii*	41	H		1								1	1				
Poaceae	Maíz	*Zea mays* L. (*)	42	H		1										1	1		
Polygonaceae	Boo	*Coccoloba cozumelensis* Hemsl.	43	T	1							1	1						
Polygonaceae	Boob	*Coccoloba spicata* Lundell (+)	44	T	1				1	1	1	1	1						
Polygonaceae	Tsi tsilche	*Gymnopodium floribundum* Rolfe (+)	45	T	1			1	1	1	1	1							
Primulaceae	Sak loo	*Ardisia escallonioides* Schltdl. & Cham. (*)	46	T	1								1	1	1				
Rhamnaceae	Chin tok	*Krugiodendron ferreum* (Vahl) Urb.	47	T	1			1	1										
Rubiaceae	Chakte cok	*Cosmocalyx spectabilis* Standl.	47	T		1									1				
Rubiaceae	Chak sabakche	*Exostema caribaeum* (Jacq.) Roem. & Schult. (*, +)	48	T		1							1						
Rubiaceae	Kuk chel	*Machaonia lindeniana* Baillon (+)	49	T	1						1	1	1	1					
Sapindaceae	Guayun kox	*Exothea diphylla* (Standl.) Lundell (+)	50	T	1				1	1									
Sapindaceae	Guayun	*Melicoccus oliviformis* Kunth ssp. *oliviformis* (+)	51	T			1	1	1	1	1	1							
Sapindaceae	Kolok	*Talisia floresii* Standl. (+)	52	T	1				1	1	1		1	1					
Sapindaceae	Kan chunub	*Thouinia paucidentata* Radlk. (+)	53	T			1	1	1	1	1	1		1	1	1	1	1	
Sapotaceae	Zapote	*Manilkara zapota* (L.) P. Royen (+)	54	T		1								1	1				
Sapotaceae	Zapotillo	*Pouteria reticulata* (Engl.) Eyma ssp. *reticulata*	55	T		1			1										

Species shared with Porter-Bolland [30] (+) and Chemas and Rico-Gray [37] (*). *CN*-*N* collector name and number, *MECM* M.E. Coh-Martínez, *1* present, *N* nectar, *P* pollen, *GH* growth habit, *T* trees, *S* shrubs, *H* herbs, *C* climbers, *L* lianas

### Flowering phenology

The blooming calendars of the plants of MF were established based on the information provided by the local beekeepers through the comprehensive interviews conducted. Subsequently, the same species were searched for their floral calendars based on the collections made in the field and with the information described on the labels of botanical specimens deposited in the collections of the CICY (Centro de Investigación Científica de Yucatán, A.C.) herbarium, and with records from the database of the global information network on biodiversity [20]. These floral calendars along with the distribution patterns of the species in the YP corroborated the knowledge of local beekeepers, and at the same time helped us to understand how botanical assemblies can be made to determine appropriate flora to increase the production of flowers during the seasons of acute food shortage for the local honeybees. The flowering months recorded for each honeybee species were classified based on the dry, rainy, and “nortes” (winds from the north) seasons that are recognized in the YP agro-climatic region. These seasons also correspond to the different stages of the local beekeeping cycle, as harvest (January to May), post-harvest (June to September), and pre-harvest (October to December) periods [12].

## Results and discussion

### Beekeepers and apiaries

**Fig. 2:**
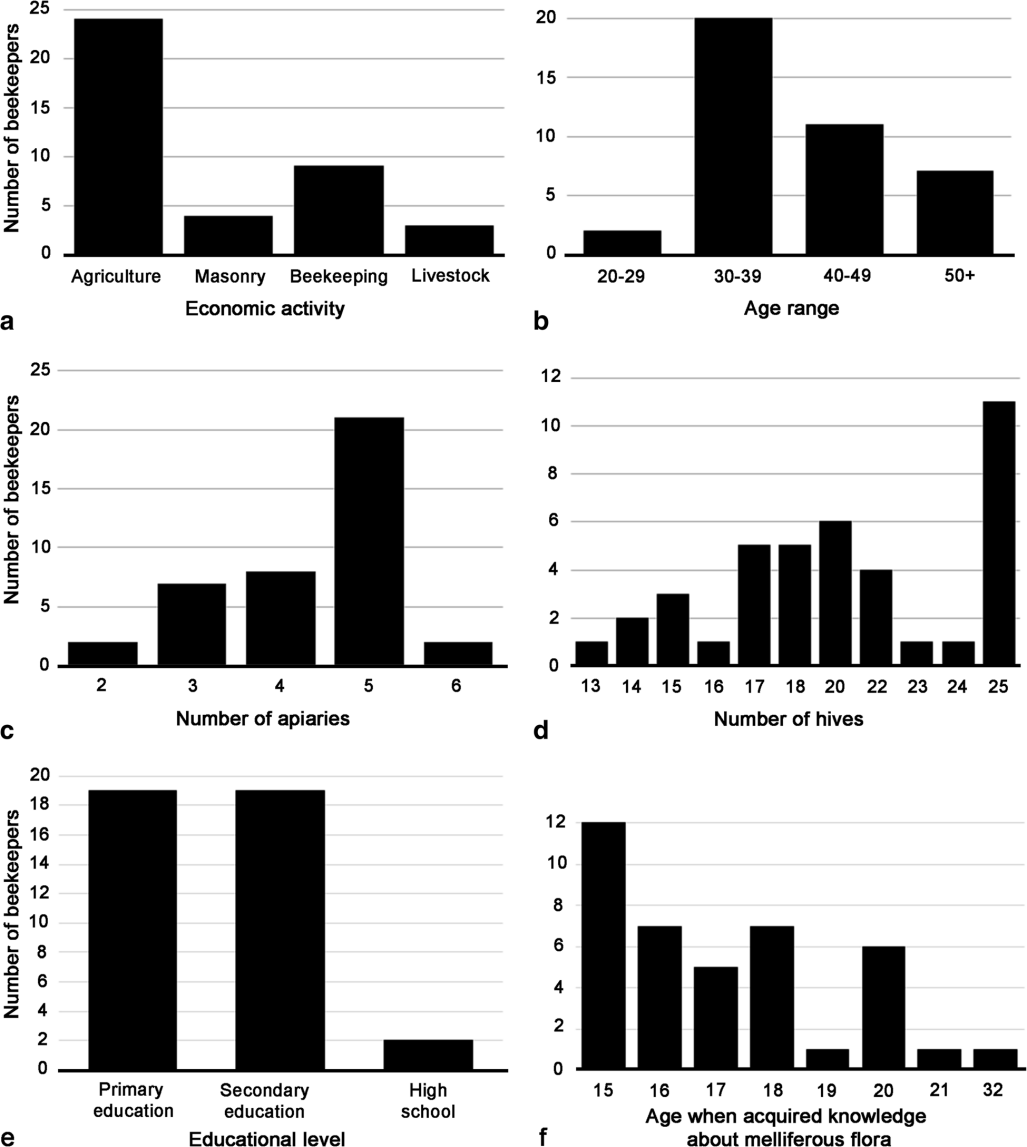
Sociodemographic characteristics, productivity, and knowledge of melliferous flora of beekeepers of the community of Xmabén, Hopelchén, Campeche, Mexico. **a** Main economic activities of beekeepers. **b** Age range of beekeepers. **c** Number of apiaries per beekeeper. **d** Number of hives per beekeeper. **e** Level of education of each beekeeper. **f** Age when acquired the knowledge about melliferous flora

Beekeeping in the community of Xmabén is carried out exclusively by men between 20 and 50 years of age and mainly with basic education. In this community, about 30% are engaged in beekeeping via parental inheritance, and the remaining 70% are involved by establishing apiaries using traditional low investment indigenous practice close to protected forests for naturally sustaining their hives. This indigenous method consists of documenting floral calendar of the MF in the area where the apiary will be established. It has been also considered that there are no apiaries to avoid competition due to availability of secondary roads and water sources (Echazareta 2010). Only 22.5% of beekeepers are dedicated to apiculture exclusively, and 77.5% practice it as a secondary economic activity due to higher financial returns from alternative livelihoods like agriculture (60%), masonry (10%), and livestock management (7.5%) (Fig. 2a).

Magaña-Magaña et al. [21] based on their socio-economic study of beekeeping in Yucatán, Mexico concluded that beekeeping is a secondary activity to agriculture due to the limited capacity of honey production and lower economic returns. Furthermore, they also noted that the high average age (49 years) and the low level of education are important factors that have been influencing lack of competitiveness and innovation in the local apiculture industry, slowing down the economic opportunity. Martínez-Puc et al. [22] reported that beekeeping is a secondary activity (2% practice it exclusively and 98% as a complementary activity) across several municipalities of Campeche due to the fact that local beekeepers depend exclusively on the availability of local MF for feeding their honeybees that varies through the year depending on the types of available MF and precipitation levels, hampering consistent high-quality honey production.

The average age of Xmabén beekeepers is 39 with 50% in the range of 30–39 years, 21% between 40 and 49 years, 7% above 50 years, and 2% between 20 and 29 years (Fig. 2b). Beekeepers over the age of 30 have 13–25 beehives and minors at this age have 23–25 beehives. The high number of hives for those under 30 is due to the fact that they have received advanced training from different government organizations (e.g., Secretariat of Agriculture, Livestock, Rural Development, Fisheries and Food (Spanish: Secretaría de Agricultura, Ganadería, Desarrollo Rural, Pesca y Alimentación; SAGARPA); Secretariat of Rural Development (Spanish: Secretaría de Desarrollo Rural; SDR)) for proper management of bee hives and disease. Unfortunately, such trainings did not provide information regarding the importance of the local MF in feeding their bees, unlike older beekeepers with traditional beekeeping knowledge. In contrast, beekeepers over 30 years using their indigenous knowledge acquired over generations often have around 25 beehives. Magaña-Magaña et al. [21] reported that beekeepers on an average aged 49 years in Yucatán, Mexico, have a greater number of hives because of their specialized indigenous knowledge (e.g., 48 years with 1–20 hives, 49 years with 21–50 hives, 53 years with 51 beehives). However, traditional beekeepers with low income have limited knowledge of modern beekeeping techniques (e.g., bee nutrition, alternative control of varroa, change of queen bees, and genetic improvement). Therefore, it is important to disseminate indigenous beekeeping knowledge successfully from older to younger generation of local beekeepers. Government training programs for local beekeepers need to cover training on local MF, and how to use this natural resource to supplement honeybee nutrition for increasing their average honey production and profit margins.

**Fig. 3:**
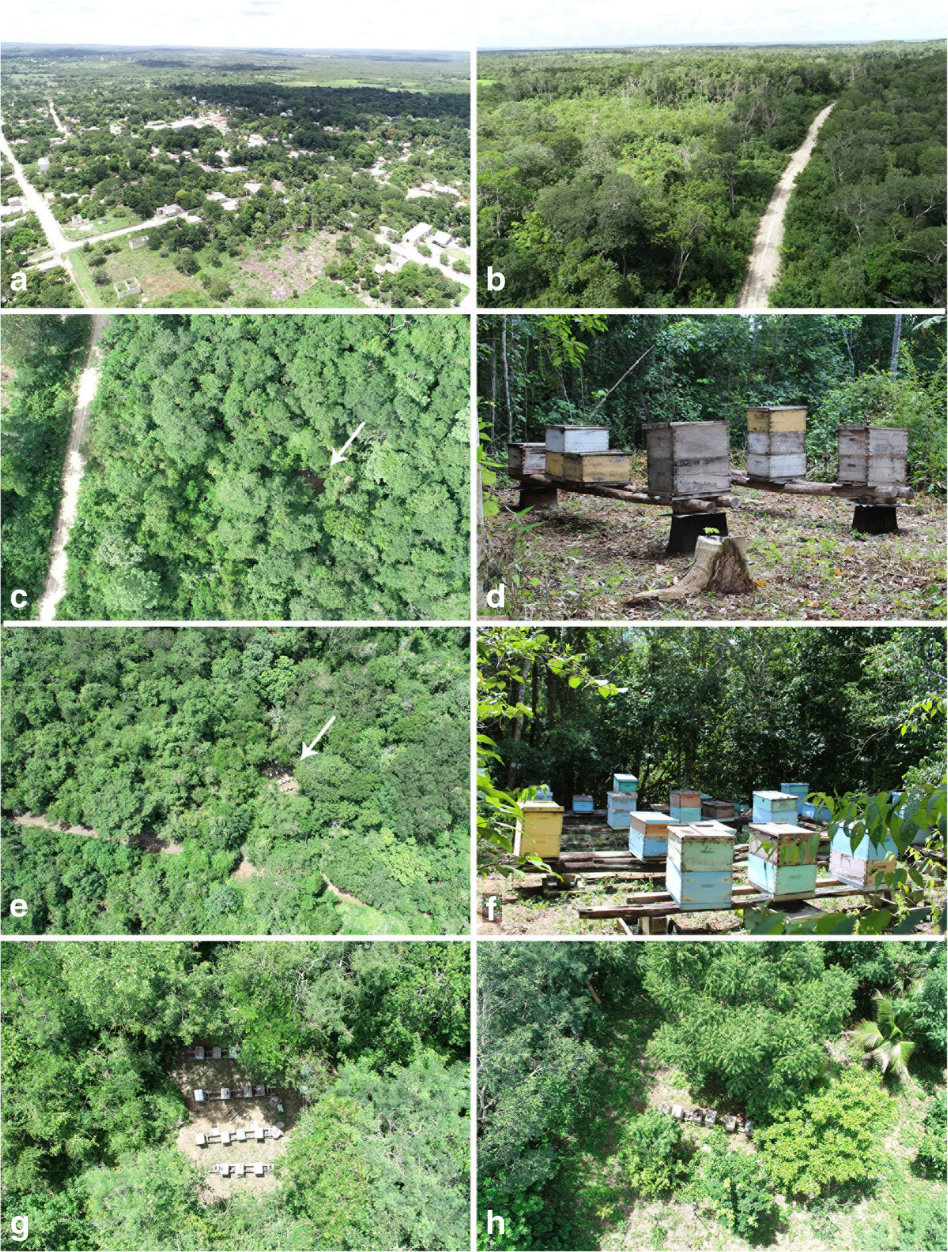
Vegetation and location of apiaries in the community of Xmabén, Hopelchén, Campeche, Mexico. **a** Community of Xmabén. **b** Dirt roads in the medium stature tropical forest (MSTF). **c**, **e** Apiaries in the MSTF and near roads. **d**, **f**, **g** Apiaries. **h** Apiaries near the community

The average number of apiaries per beekeeper in Xmabén is quite high (4.35) (Fig. 2c) when compared with beekeepers from other YP communities for example, 2.27 in the west and northwest of Campeche [23] to 2.6 apiaries [21] in other areas of Yucatán. This is mostly due to the fact that Xmabén apiaries are located closer to the agricultural production areas and hence can be visited by the beekeepers quite easily (Figs. 1 and 3). Porter and Ellis [16] previously recorded 41 apiaries in Xmaben, of which 31 were found near the village and in secondary vegetation and agriculture areas (at a distance of 1–4 km in a straight line). The remaining apiaries were recorded at a distance of 7–13 km in MSTF (Fig. 1). However, based on the 40 apiaries registered in this study (Figs. 1 and 3), it can be noted that currently only six of these apiaries are close to the town. These were moved 1 km away due to increase in local human population and since they represented a threat to the population due to possible bee stings. Only one apiary was recorded in the low flood forest (6 km distance) and the remaining 33 were established within 8–16 km in the conserved MSTF vegetation areas (Figs. 1 and 3). Most of the apiaries were established approximately a decade ago, mainly in the MSTF (Fig. 2), due to danger associated with apiaries located close to human settlement and better access to MF for feeding the bees. The beekeepers of Xmabén have large areas of communal land with MF vegetation, where they have established several apiaries. These vegetation areas are protected because community is located in an area bordering the buffer zone of the Calakmul Biosphere Reserve (Fig. 1).

At the community level, the average number of hives registered per apiary was 20.22 (27.5% of beekeepers have 25 hives per apiary) (Fig. 2d). This is similar (20.26) to that registered in other communities of Campeche (Campeche, Champotón, and Hopelchén) [23]. However, when compared to averages with other places in Mexico where the beekeeping activity is much more profitable, the value is comparatively lower. For example, in Jalisco, there is an average of 335 beehives per producer, Veracruz with 88 hives per producer, and Yucatán with 54.34 beehives per producer [24]. The states with the highest honey production in Brazil (11th country in production) with tropical flora, small producers have 5–20 beehives, medium producers of 21–50 beehives, and large producers have more than 50 beehives [25]. The low number of hives per beekeeper registered in Xmabén represents a typical example of a rural community with traditional apicultural techniques with low productivity and poor economic returns for the local beekeepers.

The educational level of local beekeepers is mainly basic education, with 47.5% having primary education, 47.5% with secondary education, and only 5% completing high school (Fig. 2e). The low level of schooling demonstrates that producers continue to use traditional practices. It is important to note that the younger people in the YP have undergone advanced training provided by SAGARPA, and the management of their hives has substantially improved. For example, in Saudi Arabia, around 40.7% of the local beekeepers have higher education. This education is an important factor empowering them to utilize modern apicultural practices for increasing honey production with higher financial returns [26]. Higher educational background also helps the beekeepers in achieving better control of bee diseases and bee nutrition [21, 22].

Several authors have pointed out that the average age of beekeepers is closely related to the level of their education and technology [21, 22]. In recent years, generational change has been encouraged to increase the productivity of apiaries in Mexico; that can be observed in the community of Xmabén, where about half are young people dedicated to beekeeping activities with high number of local apiaries. However, these governmental programs (e.g., SAGARPA) should include training on the MF and helping local beekeepers to be aware of modern apicultural practices to make their business more profitable. We also confirm the claims made by earlier authors [3, 7, 21, 22] that beekeeping in the YP is an activity secondary to agriculture, based on our own investigation. Despite these considerations and the lack of economic resources that limit innovations, there is an economic potential for improving the life and business of the beekeepers in Xmabén.

### Melliferous flora

The indigenous knowledge on MF by the Xmabén beekeepers can be categorized as follows: acquired across different generations (42.5%), shared knowledge of other older beekeepers (25%), empirical knowledge (20%), and through government programs such as SAGARPA (12.5%). The indigenous knowledge include MF along with their patterns of distribution and frequency in a particular area, the nature and type of vegetation, growth habits, flowering periods, together with environmental factors (precipitation and temperature) of the area to better understand the availability of nectar and pollen for sustenance of bee colonies and honey production [27, 28]. This knowledge about MF serves as a tool for beekeepers, since it allows them to have better management of their apiaries, decide when they should supplement bee nutrition, or change their apiaries to locations with adequate MF for the bees to forage for pollen and nectar contributing toward quality honey production [29].

Around 60% of local beekeepers began to acquire this knowledge on MF when they were teenagers (15–17 years, Fig. 2f) and had to accompany their parents to beekeeping and agriculture, while 40% of beekeepers acquired it as adults (18–32 years, Fig. 2f), when beekeeping has already become a part of their regular activities for economic sustenance. The knowledge of MF that beekeepers have in other communities of YP is transferred from one generation to another, and is important for them, since they depend exclusively on this information for the feeding their bees [30]. In other parts of the world (e.g., Ethiopia, India, Morocco and Nepal), where beekeeping is practiced with limited economic resources, several authors have reported that the knowledge of FM for a given area is absolutely important for beekeepers for feeding their bees [28, 31–33].

### Diversity and growth habits

Based on the information provided by the 40 beekeepers from the Xmabén community, 56 MF taxa (50 species along with 3 subspecies and 3 varieties) were recorded that are distributed across 26 plant families representing 50 genera. This represents 38% of the local MF, since 146 species in 35 families and 101 genera has been previously reported for the state of Campeche [34]. However, this diversity of MF estimated by Porter-Bolland [34] for Campeche is low; if all studies of apicultural flora of region are integrated together with that of the YP, the diversity of MF present in Campeche could be of approximately 792 taxa (Cetzal-Ix et al. in prep.). Hence, the MF for Xmabén would represent 7% of that region, based on the information provided by the beekeepers as mentioned above.

**Fig. 4:**
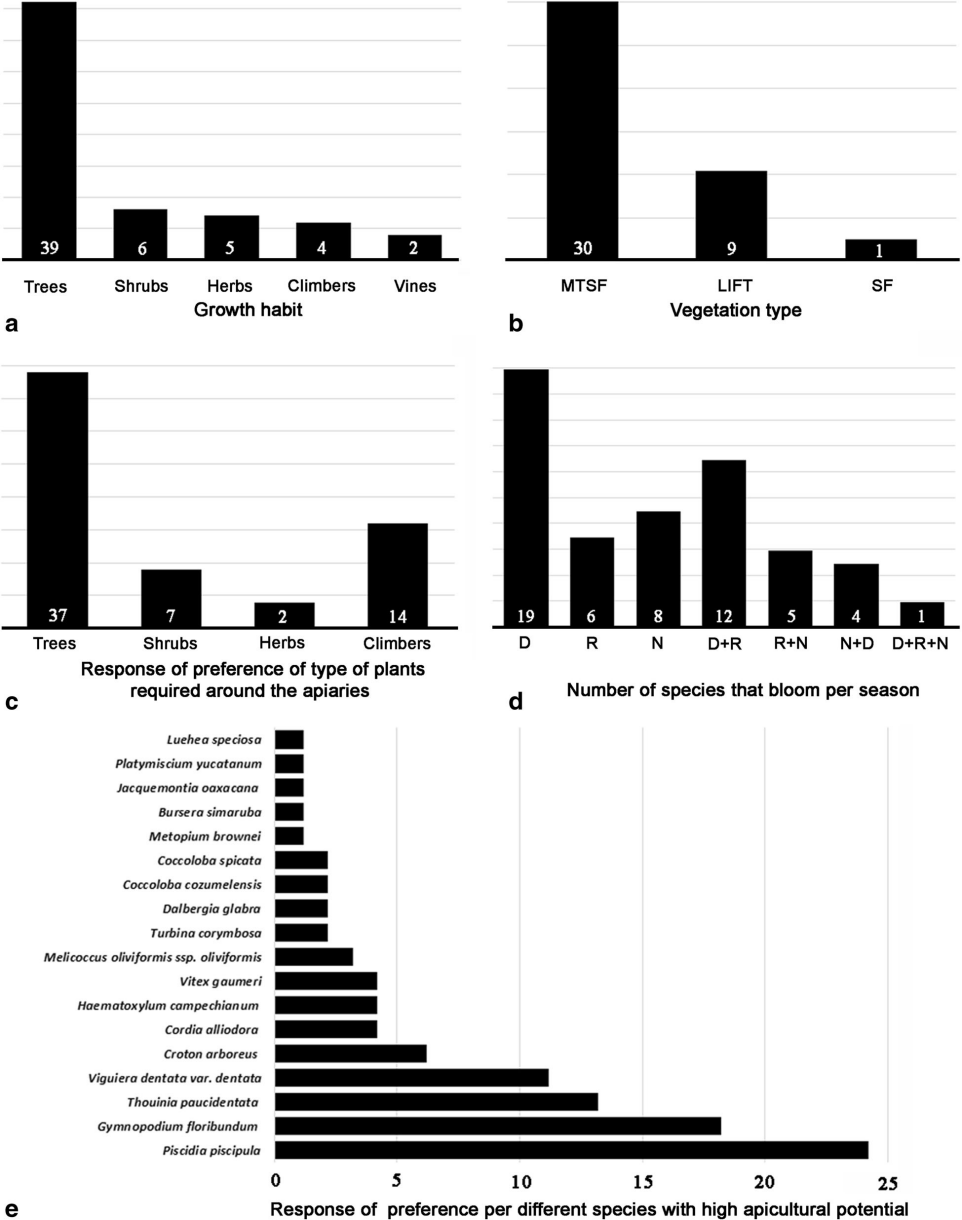
Melliferous flora in the community of Xmabén, Hopelchén, Campeche, Mexico. **a** Number of species per growth habit. **b** Number of response by beekeepers interviewed on the type of vegetation where they established their apiaries. **c** Number of response by beekeepers interviewed on type of plants required around the apiaries for feeding the bees. **d** Number of species that bloom per season. **e** Response by beekeepers interviewed for different species with high apicultural potential in Xmabén, Hopelchén, Campeche, Mexico. Vegetation type: *MTSF* medium stature tropical forest (MSTF), *LIFT* low inundated tropical forest, *SF* secondary forest. Regional season: *D* dry, *R* rainy, *N* nortes

The low diversity of plants of MF registered in Xmabén is possibly due to the fact that beekeepers’ knowledge of local flora focuses mainly on tree species (Fig. 4a, Table 1), which are part of their forest use (commercialization of woods). In addition, most of the tree species identified by beekeepers (Table 1) are typical and abundant species in the MSTF in the YP [35] and represent predominant type of vegetation for the local community and where they have established their apiaries (Fig. 4b). Nevertheless, there are floristic studies of MSTF for areas near Xmabén, where other species have been recorded that are considered as MF for the YP region [35, 36].

On the other hand, when diversity is compared with other beekeeping studies for the same area or region, the number of species found in Xmabén is high. For example, Porter-Bolland [30] recorded 100 species in 67 genera and 31 families for 8 communities in the Mountain zone (Chanchen, Chunek, Nuevo Chan Yaxche, Pachuitz, Ukum, Xkanha, Xmabén, and Xmejía). This particular area has vegetation of MSTF, low evergreen forest, low inundated tropical forest, and patches of secondary vegetation with agriculture. With this study, Xmabén shares 50% (28) of the species, and the other 50% represents unregistered species of MF for the area (Table 1). Chemas and Rico-Gray [37] recorded 36 taxa in 34 genera and 19 families in the community of Tixcaltuyub, Yucatán in a medium stature tropical forest; with this other study, it shares 30% (17) of the species (Table 1). The flora shared among these studies refers to broad species of distribution and are found in most types of vegetation of the YP (Table 1).

The information concerning the growth habits provided by the beekeepers of Xmabén indicates that the trees are more abundant, followed by shrubs, herbs, climber plants, and lianas, with 39 (70%), 6 (10%), 5 (9%), 4 (7%), and 2 (4%) species, respectively (Fig. 4a). The high number of tree species of MF registered in Xmabén coincides with other studies of the same area and for the region. For example, Porter-Bolland [30] for the Mountain zone (Campeche) recorded 101 taxa; the most abundant growth habit was the trees, followed by shrubs, climbers, and lianas, with 83 (82%), 10 (10%), 5 (5%), and 3 (3%) species, respectively. On the other hand, Chemas and Rico-Gray [37] reported 33 taxa for Tixcaltuyub (Yucatán); of these, the most abundant was the trees, followed by shrubs, herbs, climbers, and lianas, with 16 (48%), 10 (30%), 4 (12%), 2 (6%), and 1 (3%), respectively.

This pattern of dominance of the arboreal strata is more or less similar when analyzing studies that partially include MF at a regional level. In this respect, Carnevali et al. [8] in a floristic study of the YP reported 99 taxa of MF, of which 42 species are trees, 36 herbs, 11 shrubs, 6 lianas, and 4 climbers. While Alfaro-Bates et al. [38] reported 93 taxa that included 36 trees, 17 shrubs, 13 herbs, 5 climbers, 2 vines, and 2 palms. However, when a study is analyzed that includes in its entirety the MF at YP level, the herbaceous strata is the one with the highest number of species, for example, Arellano et al. [19] reported 992 taxa, 249 correspond to herbs, 226 shrubs, 158 trees, 67 climbers, 60 lianas, and 11 palms.

The largest number of tree species recorded in Xmabén is related to the knowledge of the local beekeepers, who focus primarily on the trees that bloom during most of the year (dry and rainy season) (Fig. 4c), and particularly during the dry season when the maximum honey happens. But if a complete study of the Xmabén flora is conducted, it is probable that the greatest number of species of MF will be herbaceous and climbers. Although they bloom mainly during rainy season, when the honey produced has a high degree of humidity that affects the quality and corresponding price. However, they can contribute in this season when there is a shortage of food for the local honeybees.

### Flowering phenology

With respect to the information on the phenology of MF species provided by local beekeepers in Xmabén, there were 19 species that bloom in the dry season (harvest season), 6 in the rainy season (post-harvest), and 8 in the rainy season and “nortes” (winds from the north) season (pre-harvest). In addition, there were 12 species that bloom in dry-rains, 5 in rains-nortes, 4 species in nortes-dry, and only 1 species that blooms in all the seasons of the year (*Thouinia paucidentata*) (Fig. 4d, Table 1). On the other hand, when the floral calendars for these same species were analyzed based on field and herbarium records at the YP level, the data indicate that most of the species bloom throughout the year and are not exclusive to a particular season, with some exceptions where the flowering patterns agree (e.g., *Bursera simaruba*, *Exothea diphylla*, *Jacquemontia oaxacana*, *Melicoccus oliviformis* ssp. *oliviformis*, and *Piscidia piscipula*), particularly the dry season species.

**Fig. 5:**
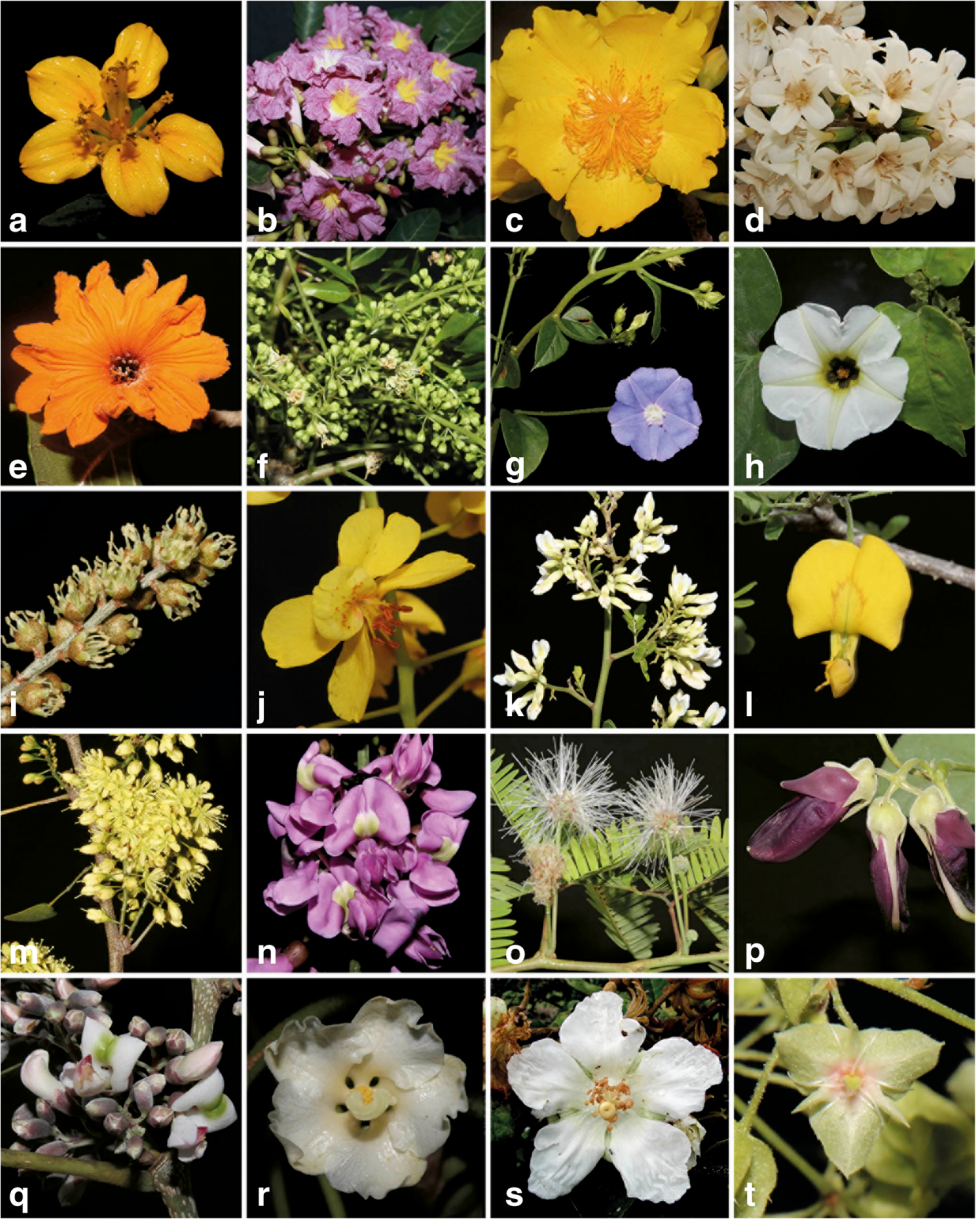
Some species of melliferous flora registered in Xmabén, Hopelchén, Campeche, Mexico. **a** *Viguiera dentata var. dentata*. **b** *Tabebuia rosea*. **c** *Cochlospermum vitifolium*. **d** *Cordia alliodora*. **e** *Cordia dodecandra*. **f** *Bursera simaruba*. **g** *Jacquemontia pentantha*. **h** *Turbina corymbosa*. **i** *Croton arboreus*. **j** *Caesalpinia gaumeri*. **k** *Dalbergia glabra*. **l** *Diphysa yucatanensis*. **m** *Haematoxylum campechianum*. **n** *Lonchocarpus punctatus*. **o** *Lysiloma latisiliquum*. **p** *Mucuna pruriens*. **q** *Piscidia piscipula*. **r** *Hampea trilobata*. **s** *Gymnopodium floribundum*

However, the data obtained from the phenology based on field and herbarium records only allow us to know the month of flowering but not when its flowering peaks occur. In contrast, the observations of local beekeepers are based on when the flowering peaks occur as part of the regular beekeeping cycle for pre harvest, harvest, and post-harvest periods. For this reason, several authors [11, 30, 37] in other studies on local MF based on interviews with the beekeepers have identified the main species in the three phases of the local beekeeping cycle, and those are the species that are required around their apiaries for stable honey production. Their observations are very similar to the species indicated by Xmabén beekeepers in the current study (Figs. 4e and 5).

**Fig. 6:**
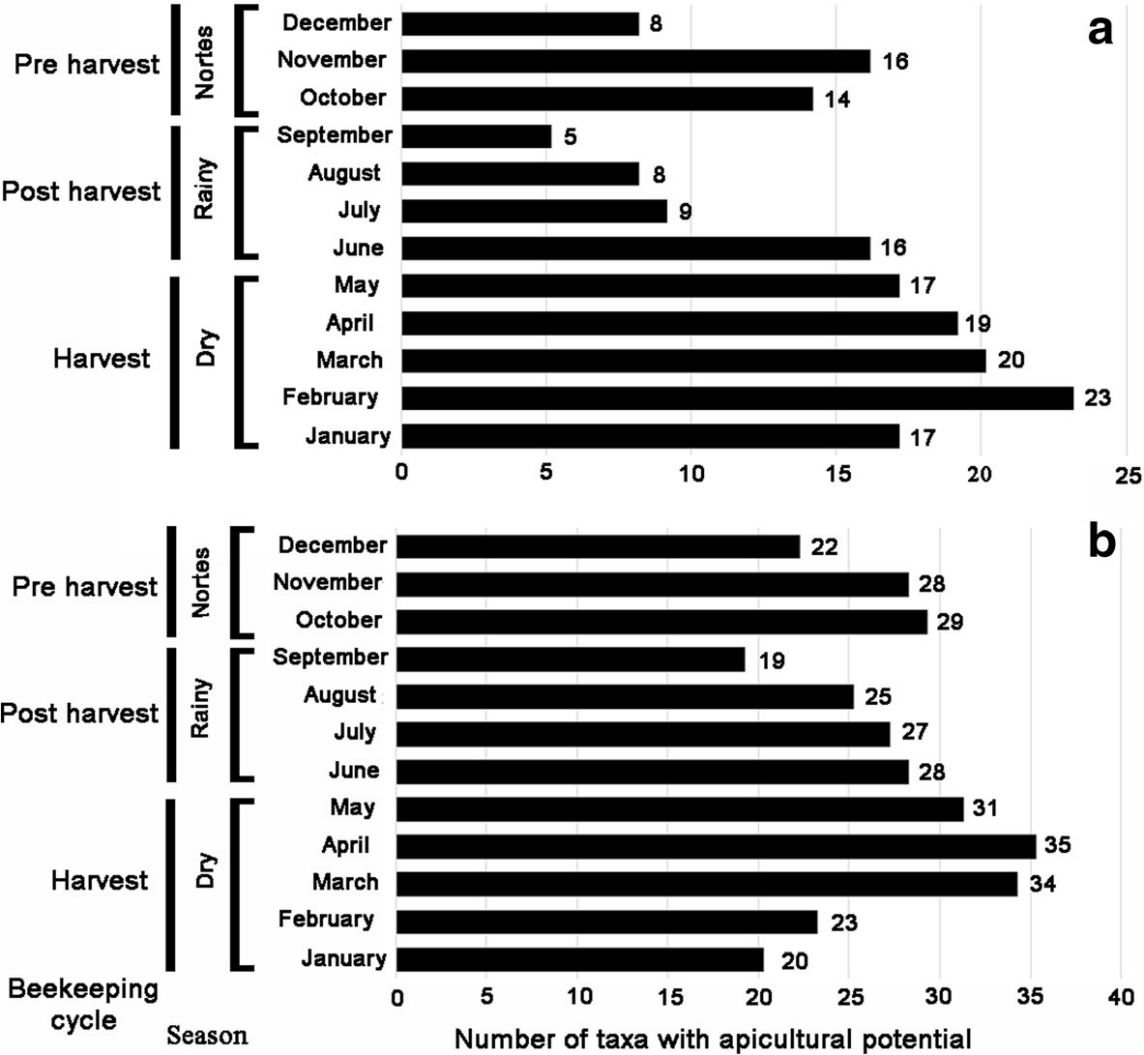
Floral calendar of melliferous flora in Xmabén, Hopelchén, Campeche, Mexico. **a** Based on interviews. **b** Based on field records and specimens of herbarium

Based on the number of species that bloom per month according to the information provided by the local beekeepers, it can be indicated that the greatest diversity of MF species flower from January to June, in the dry season, and at the beginning of the rainy season, when honey is harvested (Fig. 6a). Subsequently, from July to September, there is a decline in flowering (Fig. 6a) during the rainy season, when there is the production of wet honey, considered to be inferior in quality and low in price [10, 11]. This poor-quality honey is due to the fact that the humid climate favors the fermentation of honey, reducing the storage time and changing the organoleptic properties of the honey produced [12].

In addition, the rainy season is considered by some researchers as the “*crisis time for the bees*” due to lack of food and nutrition [7, 30], since the colonies of honeybees decrease or they abandon their nests in search of nectar and/or pollen [7, 30]. Also, the honeybee colonies are prone to attacks by various pests, diseases, and predators [7]. According to the local Xmabén beekeepers, the main problem associated with this period of food shortage in bees is the plague of the small hive beetle (*Aethina tumida* Murray) and the varroosis disease caused by the mite *Varroa destructor* Anderson & Trueman. Finally, from October to December, the nortes season begins, and the number of plant species in flowering increases again (Fig. 6a); this stage is known as post-harvest or recovery phase [7]. In this phase, a wide variety of climbing plants are found in flowering, among them the family Convolvulaceae [11, 38] is most abundant across the Xmabén community.

When comparing the number of species that bloom in each of the seasons of the year between the observations of the local beekeepers versus the botanical registers of field and herbarium at regional level, it can be mentioned that we noted a more or less similar pattern (Fig. 6a, b). The greatest diversity of species blooms in the dry season, later there is a decrease in flowering during the rainy season, and again an increase in the nortes season. This previous pattern of the number of species that flourish in each of the seasons is repeated in several studies analyzed for the region [11, 30, 38], and this is associated with the beekeeping cycle for local honey production.

The local MF provides the beekeepers with great economic benefits with respect to quality honey production in marketable amounts. The flowers of the plants have nectar or pollen or both in the same flower. The honeybees collect the nectar and transform it into honey. The pollens collected are fed to their youngs, an essential activity necessary for the continuous propagation and future development of the hive [7]. In Xmabén, local beekeepers pointed out that MF provide mainly nectariferous resources (25), which is the main material for the production of honey. Secondarily polliniferous resources (9) offer both nectar and polliniferous resources (17 species) for the feeding of the honeybees. In this aspect, beekeepers indicate that they depend on 18 species of MF to obtain a higher honey production (Fig. 4e).

In this work, we have highlighted the highly neglected but extremely valuable indigenous knowledge of the local beekeeping community of the YP region, pinpointing specific issues of the community of Xmabén. Our study is unique in the sense that we have narrowed down our investigation to a specific high-quality bee production area within the YP region. There is information available broadly across the Yucatan region, but there is dearth of information from credible micromanagement perspective from specific beekeeping communities, districts, and units within that region. In this context, this study humbly attempts to reduce the gap between the indigenous community-based beekeeping practices with that of modern apiculture from an international perspective.

## Conclusions

Based on the interviews conducted on the local beekeepers of the Xmabén community, it can be mentioned that they have appreciably good knowledge of their local MF, the flowering patterns, and specific flora that provide nectar and/or pollen to the local honeybees. They also are aware of the most relevant MF species that they must have around their apiaries to successfully complete the local beekeeping cycle across different seasons for quality honey production for local, regional, and international markets. Most beekeepers get honey according to what nature provides them. However, to take advantage of the maximum potential of the local vegetation and their flowering periods, the apiaries should be established in appropriate areas that has abundant trees that do not bloom all year round or during the rainy season. For this purpose, it is important to have sufficient number of local climbers and other annual herbs, so that during the rainy season the honeybees have easily available food and foraging resources and they do not need to migrate to other apiaries for their survival. The knowledge shared by the Xmabén beekeepers will allow other beekeepers in the region to have guidelines to establish strategies to keep the colonies of honeybees healthy, and with sufficient food resources during the periods of food shortage. Hence, they will be able to make higher production of high-quality honey and earn bigger profits for their business. Our work will help strengthen the integrated use of indigenous knowledge and practices of beekeeping with modern apiculture practices for enhancing local honey production and sustainable growth of the local apiculture industry.

## Abbreviations

MF: Melliferous flora
MSTF: Medium stature tropical forest
YP: Yucatan Peninsula
